# Antibiotic resistance in the opportunistic pathogen *Stenotrophomonas maltophilia*

**DOI:** 10.3389/fmicb.2015.00658

**Published:** 2015-06-30

**Authors:** María B. Sánchez

**Affiliations:** Departamento de Biotecnología Microbiana, Centro Nacional de Biotecnología, Consejo Superior de Investigaciones CientíficasMadrid, Spain

**Keywords:** *Stenotrophomonas maltophilia*, antibiotic resistance, multidrug resistance, intrinsic resistome, phenotypic resistance

## Abstract

*Stenotrophomonas maltophilia* is an environmental bacterium found in the soil, associated with plants and animals, and in aquatic environments. It is also an opportunistic pathogen now causing an increasing number of nosocomial infections. The treatment of *S. maltophilia* is quite difficult given its intrinsic resistance to a number of antibiotics, and because it is able to acquire new resistances via horizontal gene transfer and mutations. Certainly, strains resistant to quinolones, cotrimoxale and/or cephalosporins—antibiotics commonly used to treat *S. maltophilia* infections—have emerged. The increasing number of available *S. maltophilia* genomes has allowed the identification and annotation of a large number of antimicrobial resistance genes. Most encode inactivating enzymes and efflux pumps, but information on their role in intrinsic and acquired resistance is limited. Non-typical antibiotic resistance mechanisms that also form part of the intrinsic resistome have been identified via mutant library screening. These include non-typical antibiotic resistance genes, such as bacterial metabolism genes, and non-inheritable resistant phenotypes, such as biofilm formation and persistence. Their relationships with resistance are complex and require further study.

*Stenotrophomonas maltophilia* is an opportunistic nosocomial pathogen that has caused an increasing number of infections in recent years (Brooke, 2012). It is associated with a number of clinical syndromes, such as endocarditis, urinary infections, and respiratory infections, including pneumonia in patients with cystic fibrosis and the immunocompromised (Falagas et al., 2009; Looney et al., 2009).

*S. maltophilia* shows low susceptibility to many antibiotics, including those commonly used to treat the infections it causes. It is therefore crucial that new antibiotic targets be found, and the appearance of resistance during treatment be predicted. The analysis of resistance mechanisms and the identification of antibiotic resistance genes can help in this. Bioinformatic studies have identified genes showing homology to known antibiotic resistance genes, although their exact functions remain to be confirmed. In recent years, deep sequencing technologies have allowed the complete sequencing of two clinical *S. maltophilia* strains, K279a and D457 (Accession numbers: NC_010943.1 and NC_017671.1) (Crossman et al., 2008; Lira et al., 2012), and two environmental strains, R551-3 and JV3 (Accession numbers: NC_011071.1 and CP002986.1). The assembly of the sequences of several other strains is currently underway, and results should be available in the near future. Genome sequencing has revealed much of the genome to be conserved across different *S. maltophilia* strains (Rocco et al., 2009; Alavi et al., 2014). In addition, most of the genes associated with resistance in *S. maltophilia* have been found present in all strains examined. However, the genomes show also sequence variability, associated to normal evolution (mutation frequency) or induced in some situations. Antibiotic pressure increases the sequence variability in resistance or related genes, as regulators. The use of quinolones in *S. maltophilia* allows the appearance of mutants overexpressing efflux pumps, first SmeDEF, and when this is not present, SmeVWX. In both cases the overexpression is mainly associated to mutations in their regulators, SmeT and SmeRv, respectively (Garcia-Leon et al., 2014b).

The analysis of transposon mutant libraries allows the identification of genes which, if deleted, alter susceptibility to antibiotics, including genes that might appear to have no clear relationship with antibiotic resistance. The drawback of this type of analysis is that, since essential genes cannot be deleted, their putative involvement in antibiotic susceptibility cannot be confirmed. This methodology has, however, been successfully used with *Pseudomonas aeruginosa* and *Escherichia coli* (Girgis et al., 2009; Alvarez-Ortega et al., 2010). When *S. maltophilia* was thus examined, genes involved in biofilm formation appeared as being related to antibiotic resistance (Huang et al., 2006; Kang et al., 2015).

Transformation with genomic libraries allows the effect of gene overexpression to be investigated; genes whose overexpression (usually in heterologous microorganisms) exerts a direct effect on antibiotic susceptibility can therefore be identified. Only those genes which exert a direct effect on antibiotic susceptibility identified in this way. The use of a *S. maltophilia* D457R chromosomal DNA library allowed the identification of genes related to antibiotic resistance, and the subsequent cloning and characterization of the SmeDEF efflux pump (Alonso and Martinez, 2000; Alonso et al., 2000).

Evolution assays can provide information on the genes involved in acquired resistance. Such information could be useful for the rational design of better treatments since it might help predict the appearance of resistance.

## Antibiotic resistance mechanisms in *S. maltophilia*

The reduced susceptibility of *S. maltophilia* to antibiotics has been associated with intrinsic resistance factors common to all *S. maltophilia* strains, such as low membrane permeability, the presence of multidrug resistance (MDR) efflux pumps, antibiotic-modifying enzymes, and the quinolone resistance gene Sm*qnr* (Crossman et al., 2008; Sanchez et al., 2009) (Table 1). Resistance can also be acquired via the acquisition of mutations or resistance genes through horizontal gene transfer (HGT). Microorganisms sharing the same environment can provide these exogenous genes. It has been postulated that other unknown mechanisms may also help account for the *S. maltophilia* antibiotic resistance phenotype. The intrinsic resistome has been defined as the group of chromosomal genes involved in the intrinsic resistance present in the strains of a bacterial species prior to exposure to an antibiotic and which is not due to HGT (Fajardo et al., 2008). The intrinsic resistome involves known and unknown genes related to antibiotic resistance, which might include genes involved in cell metabolism (Olivares et al., 2013). To date, most studies have focused on classical antibiotic resistance genes, such as those coding for efflux pumps or modifying enzymes (intrinsic resistance), and on the appearance during clinical treatment of mutants showing low susceptibility to antibiotics (acquired resistance). Our knowledge of the *S. maltophilia* intrinsic resistome is, however, limited.

**Table 1:** **Summary of known antibiotic resistances genes in** ***S. maltophilia*****, antibiotic resistance profile and their role in intrinsic and acquired resistance**.

**Gene(s)**	**Product**	**Antibiotic resistance phenotype**	**Intrinsic/Acquired antibiotic resistance**	**Reference**
*smeABC*	RND-type efflux pump	Aminoglycosides, β-lactams, and quinolones	No/Yes	Li et al., 2002
*smeDEF*	RND-type efflux pump	Chloramphenicol, tetracycline and quinolones	Yes/Yes	Alonso and Martinez, 2000; Zhang et al., 2001
*smeGH*	RND-type efflux pump	Unknown	ND/ND	Crossman et al., 2008
*smeIJK*	RND-type efflux pump	Aminoglycosides, tetracycline and ciprofloxacin	Yes/Yes	Crossman et al., 2008; Gould et al., 2013
*smeMN*	RND-type efflux pump	Unknown	ND/ND	Crossman et al., 2008
*smeOP*	RND-type efflux pump	Aminoglycosides, nalidixic acid, doxycycline, macrolides	Yes/No	Lin et al., 2014a
*smeVWX*	RND-type efflux pump	Chloramphenicol and quinolones	No/Yes	Chen et al., 2011; Garcia-Leon et al., 2014b
*smeYZ*	RND-type efflux pump	Aminoglycosides	Yes/yes	Crossman et al., 2008; Gould et al., 2013
*emrCABsm*	MFS-type efflux pump	Nalidixic acid and CCCP	No/Yes	Huang et al., 2013a
*smlt0032*	MFS-type efflux pump	Unknown	ND/ND	Crossman et al., 2008
*smtcrA*	MFS-type efflux pump	Tetracycline	No/Yes	Chang et al., 2011
*smrA*	ABC-type efflux pump	Fluoroquinolones, tetracycline, doxorubicin	ND/Yes	Al-Hamad et al., 2009
*macABCsm*	ABC-type efflux pump	Macrolides, aminoglycosides and polymyxins	Yes/ND	Lin et al., 2014b
*L1*	β-lactamase	β-lactams	Yes/Yes	Hu et al., 2008; Okazaki and Avison, 2008
*L2*	β-lactamase	β-lactams	Yes/Yes	Hu et al., 2008; Okazaki and Avison, 2008
*aph (3′)-IIc*	Aminoglycoside phosphotransferase	Aminoglycosides	Yes/Yes	Okazaki and Avison, 2007
*aac (6′)-Iz*	N-Aminoglycoside acetyltransferase	Aminoglycosides	Yes/Yes	Li et al., 2003
Sm*qnr*	Pentapeptide Repeat Proteins	Quinolones	Yes/Yes	Sanchez and Martinez, 2010; Chang et al., 2011

ND not determined

Like all Gram negative bacteria, *S. maltophilia* shows low membrane permeability—the consequence of it having two cell membranes and a peptidoglycan wall. The outer membrane is an efficient barrier. Mutants showing altered outer membrane permeability or which have a different lipopolysaccharide structure show modified susceptibility to antibiotics (Vaara, 1993; Rahmati-Bahram et al., 1996).

Low susceptibility to antibiotics is often related to the presence of active efflux pumps. Such pumps have been identified in *S. maltophilia* K279a, including eight MDR efflux pumps belonging to the putative resistance nodulation cell division (RND) family, two belonging to the major facilitator superfamily (MFS), and two ATP-binding cassette (ABC) pumps (Crossman et al., 2008). In Gram negative bacteria, RND efflux pumps are composed of three proteins: an inner membrane protein, which binds the substrate, an outer membrane protein (porin), and a membrane fusion protein (MFP), which binds the outer and inner proteins in the periplasmic space. In general, the genes coding for the porin, MFP and inner protein are located in the same operon. Some exceptions in which there is no porin-coding gene have been identified. In addition, MDR efflux pumps are modulated by a regulator protein encoded by a gene located upstream and divergently transcribed from the efflux pump operon. In general, most efflux pump machinery is expressed at low levels (Li et al., 2002; Lin et al., 2014a). Overexpression is associated with low antibiotic susceptibility, and is sometimes related to mutations in regulator genes. Such mutations have been identified both *in vitro* and *in vivo* (Alonso and Martinez, 2001; Cho et al., 2012; Gould et al., 2013; García-León et al., 2015), supporting the idea that *in vitro* evolution studies may be able predict mutations appearing *in vivo* during the treatment of patients.

All the proteins of the efflux pumps SmeABC, SmeDEF and SmeVWX, which belong to the RND family, are encoded in the same operon following the typical genomic arrangement. The roles of these efflux pumps in intrinsic and acquired resistance have been extensively characterized (Alonso and Martinez, 2000; Li et al., 2002; Chen et al., 2011). SmeABC is involved in acquired resistance to β-lactams, aminoglycosides and quinolones, but has no influence on intrinsic resistance. The deletion of the *smeC* gene (porin) affects susceptibility to several antibiotics (Li et al., 2002), suggesting its possible relationship with other efflux pumps. SmeDEF is involved in both intrinsic and acquired resistance to chloramphenicol, tetracycline and quinolones, as well as acquired resistance to non-antibiotic compounds such as triclosan (Sanchez et al., 2005; Hernandez et al., 2011). SmeVWX has a role in acquired resistance to the same antibiotics (Alonso and Martinez, 2001; Zhang et al., 2001; Chen et al., 2011; Garcia-Leon et al., 2014b). In acquired resistance, the overexpression of the SmeDEF and SmeVWX efflux pumps is related to mutations in the regulators SmeT and SmeRv, respectively (Sanchez et al., 2002; Garcia-Leon et al., 2014b).

Other *S. maltophilia* efflux pumps have recently been studied, including SmeIJK and SmeYZ, which also belong to the RND family. Both have a role in intrinsic and acquired resistance, SmeJK to aminoglycosides, tetracycline and ciprofloxacin, and SmeZ to aminoglycosides (Crossman et al., 2008). In addition, their overexpression provides resistance to levofloxacin (Gould et al., 2013). Neither of these efflux pumps has a known associated porin. The efflux pump SmeOP, another RND family member, confers low susceptibility to aminoglycosides, nalidixic acid, doxycycline, macrolides and certain not antibiotic compounds, such as carbonyl cyanide 3-chlorophenylhydrazone (CCCP), crystal violet, sodium dodecyl sulfate (SDS), and tetrachlorosalicylanilide (TCS). In the acquired resistance setting, however, it provides protection only against CCCP and TCS (Lin et al., 2014a). The TolCsm porin has been associated with the SmeOP efflux pump. The *tolCsm* gene is located upstream of the *smeOP* operon, in another operon known as *smeRo-pcm-tolC*. The Δ*tolCsm* phenotype increases susceptibility to several compounds (Huang et al., 2013b), although no correlation is seen with the Δ*smeOP* phenotype. This suggests that the TolCsm porin is not exclusive to the SmeOP efflux pump (Huang et al., 2013b; Lin et al., 2014a).

Bioinformatic analyses have also identified two putative MFS-type tripartite efflux transporters (Crossman et al., 2008). One of these, *emrCABsm*, shows high homology with *emrAB* of *E. coli* (Lomovskaya and Lewis, 1992). In *S. maltophilia*, this pump is encoded by an operon of four genes that cover the three efflux pump components and a MarR-type regulator, *emrRsm*, which is expressed in the same direction. *ΔemrRsm* mutants show low susceptibility to nalidixic acid and CCCP due to the overexpression of the efflux pump, indicating *emrRsm* to act as a repressor (Huang et al., 2013a).

Although ABC-type transporters play a major role in Gram positive bacteria, they have also been found in Gram negative organisms, e.g., MsbA and MacAB in *E. coli*, VcaM in *Vibrio cholerae*, MacAB in *Neisseria gonorrhoeae*, and SmdAB in *Serratia marcescens* (Lin et al., 2014b). Two ABC efflux pumps, SmrA and MacABCsm, have also been described in *S. maltophilia* (Al-Hamad et al., 2009; Lin et al., 2014b). The SmrA pump has only been studied in the heterologous microorganism *E. coli*, in which it provides resistance to fluoroquinolones, tetracycline, doxorubicin and multiple dyes; its role in *S. maltophilia* remains unknown (Al-Hamad et al., 2009). The other ABC-type efflux pump, MacABCsm, is associated with intrinsic resistance to macrolides, aminoglycosides and polymyxins. Interestingly, Δ*macCsm* mutant bacteria show lower susceptibility to polymyxins, aminoglycosides and macrolides than Δ*macAB* mutants, suggesting that the MacABCsm efflux pump uses an alternative, still-unidentified porin (Lin et al., 2014b). This efflux pump is constitutively expressed, contributing toward oxidative and envelope stress tolerances and biofilm formation. The original function of the MacABCsm efflux pump may therefore have seen it involved in metabolism or adaptation to environmental changes (Lin et al., 2014b).

Bioinformatic analyses have predicted the existence of additional pumps. For example, the Sm*tcrA* gene, which codes for a putative MFS pump, has been associated with tetracycline resistance. Whether any other components are required to make this pump work remains unknown (Chang et al., 2011).

The presence of active extrusion mechanisms cannot, however, explain the low susceptibility of *S. maltophilia* to all antibiotics, and indeed the *S. maltophilia* genome codes for several modifying enzymes responsible for ß-lactam and aminoglycoside resistance phenotypes. *S. maltophilia* possesses two inducible ß-lactamases: L1, a Zn^2+^-dependent metalloenzyme which can hydrolyze nearly all classes of β-lactams (though not monobactams), and L2, a serine active-site cephalosporinase (Avison et al., 2001). The expression of both enzymes is regulated by *ampR* (a LysR type regulator located upstream of the L2 gene), and induced by the presence of ß-lactam antibiotics. AmpR acts as a weak repressor of the L2 gene in the absence of the inducer, and as an activator in its presence. With respect to L1, AmpR is required both for basal and induced expression (Lin et al., 2009). Other expression-regulating mechanisms also influence one or the other of the two enzyme genes, without affecting the regulated expression of the other. However, the exact mechanism of this additional regulation system remains to be elucidated (Avison et al., 2002; Okazaki and Avison, 2008). Finally, the expression of these enzymes in *S. maltophilia* is also subject to a complex regulation network. The deletion of the *ampN-ampG* operon, which encodes a permease transporter, prevents the induction of β-lactamases (Huang et al., 2010), while the inactivation of *mrcA*, which is predicted to encode penicillin-binding protein 1 (PBP1a), or of *ampD_1_*, which encodes a cytoplasmic N-acetyl-muramyl-L-alanine amidase, causes the hyperproduction of L1/L2 β-lactamase (Yang et al., 2009; Lin et al., 2011).

*S. maltophilia* also encodes two aminoglycoside modifying enzymes, conferring low susceptibility to aminoglycoside antibiotics (with the exception of gentamicin). Gene *aph* (3′)-*IIc* encodes an aminoglycoside phosphotransferase (Okazaki and Avison, 2007), while *aac (6′)*-*Iz* codes for an N-aminoglycoside acetyltransferase. The latter has three alleles—*aac(6′)*-*Iz, aac(6′)*-*Iaz* and *aac(6′)*-*Iam—*that show more than 80% similarity (Li et al., 2003; Tada et al., 2014). The presence of other inactivating enzymes (2′N-acetyltransferase, streptomycin 3″phosphotransferase/kinase, spectinomycin phosphotransferase, and chloramphenicol acetyltransferase) might be responsible for the susceptibility phenotype of *S. maltophilia* (Crossman et al., 2008). More studies are needed, however, to confirm their function.

*S. maltophilia* shows low susceptibility to synthetic antibiotics such as quinolones. Mutations in topoisomerases, the quinolone target, have been related to the main quinolone resistant mechanism in all bacteria. However, no topoisomerase mutations have ever been identified in *S. maltophilia* (Ribera et al., 2002; Valdezate et al., 2002; Garcia-Leon et al., 2014b). In contrast, *S. maltophilia* quinolone resistance is owed to efflux pumps (Alonso and Martinez, 2000; Li et al., 2002; Chen et al., 2011; Garcia-Leon et al., 2014b) and to the quinolone resistance protein SmQnr. This protein has been associated with both intrinsic and acquired resistance in *S. maltophilia* by a still unknown mechanism (Sanchez and Martinez, 2010; Chang et al., 2011). Qnr forms a dimer with a structure similar to double stranded DNA (Vetting et al., 2006; Xiong et al., 2011). Then, it has been proposed that SmQnr would bind topoisomerases protecting them, similarly to what was described for Qnr encoded in plasmid (Tran and Jacoby, 2002; Tran et al., 2005).

There is limited information on the antibiotic resistance mechanisms operating in clinical *S. maltophilia* strains. To date, overexpression of efflux pumps SmeABC, SmeDEF and SmeVWX, and the presence of class 1 integrons with antibiotic resistance genes have been associated with low susceptibility in clinical strains (Alonso and Martinez, 2001; Liaw et al., 2010; Cho et al., 2012).

## Phenotypic resistance

In addition to the antibiotic resistance genes described, microorganisms may possess a non-inheritable resistance mechanism known as phenotypic resistance. Some (or indeed all) of a bacterial population, may temporarily appear less susceptible to an antibiotic, without the appearance of any genomic differences. The factors responsible for phenotypic resistance might be good targets for novel treatments. However, our knowledge of the genes responsible for phenotypic resistance remains limited.

Biofilms are complex structures composed of an exopolysaccharide matrix, DNA and proteins, in which bacteria lie. They often affect clinical equipment such as catheters and other devices, from which they can be difficult to remove. The reduction in susceptibility to antibiotics afforded by biofilms is due to the difficulty of making the antibiotic come into contact with the bacteria, and these bacteria having a metabolic status different to that of their non-biofilm counterparts (a consequence of differences in the availability of nutrients and oxygen, etc.).

Different factors involved in biofilm formation have been studied in *S. maltophilia*. The deletion of different genes related to the regulation and structure of flagella, and to exopolysaccharide synthesis, affects biofilm formation (Huang et al., 2006; Kang et al., 2015). Other biofilm components, such as extracellular DNA, have been analyzed in microorganisms such as *P. aeruginosa* and *Salmonella enterica* (Mulcahy et al., 2008; Johnson et al., 2013), but the literature contains no information for *S. maltophilia*.

Bacteria showing low susceptibility to antibiotics but which are genetically identical to the susceptible strain can become persistent. Different genes involved in *E. coli* persistence include those coding for toxin/antitoxin systems and the PhoU regulator (Olivares et al., 2013). Although *S. maltophilia* persistence increases in chronic infections (Brooke, 2012), the mechanisms responsible for this phenotype remain unknown.

Post-transcriptional and post-translational regulation or modification can also alter antibiotic resistance. In *S. maltophilia*, however, little is known about this kind of regulation. Non-coding small RNAs (sRNA) and the RNA-binding Hfq protein have been related to post-transcriptional gene expression, and in *E. coli* an sRNA and Hfq have been associated with antibiotic susceptibility (Moon and Gottesman, 2009). In *S. maltophilia*, 60 sRNA candidates and a *hfq* gene have been identified, and a *Δhfq* mutant has been associated with changes in antibiotic susceptibility, biofilm production, motility and the expression of several sRNAs (Roscetto et al., 2012). However, further studies are needed to determine the role of sRNAs in antibiotic susceptibility.

## Intrinsic resistome

The study of the intrinsic resistome could provide novel antibiotic targets and help predict events during treatment. The intrinsic resistome has been studied in *P. aeruginosa* (Fajardo et al., 2008) and *E. coli* (Tamae et al., 2008; Girgis et al., 2009; Liu et al., 2010), and many genes whose deletion affects the antibiotic susceptibility phenotype have been identified. Recently, the screening of an *S. maltophilia* insertion mutant library identified *smeT*, which codes for a well-known regulator of the SmeDEF efflux pump, and *mutS*, which has a role in the DNA mismatch repair system. Other genes, e.g., the 23S gene, with no obvious role in antibiotic susceptibility may however influence it (Bernardini, 2014). Further analyses are required to precisely determine the role and mechanism of action of these genes in *S. maltophilia* antibiotic resistance.

The presence of this great number of influencing genes and antibiotic resistance mechanisms in *S. maltophilia* renders the treatment of its infections complicated. Several antibiotics are currently in use, including synthetic antibiotics and antibiotic combinations, that help prevent the appearance of resistant mutants. A trimethoprim/sulfamethoxazole (cotrimoxazole) combination is used as a last treatment option. Cotrimoxazole resistance in *S. maltophilia* has been associated with the genes *sul1* and *sul2*. These have been linked to the presence of class 1 integrons in plasmids in the main, but also in the chromosomal genome (Barbolla et al., 2004; Toleman et al., 2007). The presence of these genes, however, cannot explain all the cases of cotrimoxazole resistance recorded. Porin TolCsm deletion also increases cotrimoxazole susceptibility (Huang et al., 2013b), but further studies are required to determine whether other porins or efflux pumps are also involved.

How to avoid the antibiotic resistance? This has been a problem since the beginning of antibiotics use. The search of new antibiotics, inhibitors of efflux pumps (Leitner et al., 2011), new targets among genes form intrinsic resistome or use of combination of known antibiotics, as trimethoprim/sulfamethoxazole (described above), are some of the new strategies to avoid not only resistant strains but also the appearance of resistant mutants.

In summary, *S. maltophilia* possesses a great many antibiotic resistance mechanisms. Most of the genes involved were present in *S. maltophilia* before any use of antibiotics. For example, the efflux pump SmeDEF is associated with the ability of *S. maltophilia* to colonize plants, and its regulator SmeT is induced by plant-produced flavonoids (Garcia-Leon et al., 2014a); thus, the main function of the genes encoding them is unlikely to be the provision of antibiotic resistance. Other mechanisms might appear in the future, depending on antibiotic pressure, the emergence of mutations, and gene acquisition events. While resistance may benefit bacteria in the presence of antibiotics, in other situations it could impair growth, as has been described for the overexpression of the efflux pump SmeDEF (Alonso et al., 2004). Further, the fitness cost of acquired resistance in *S. maltophilia* determines whether new mechanisms are kept.

### Conflict of interest statement

The author declares that the research was conducted in the absence of any commercial or financial relationships that could be construed as a potential conflict of interest.
